# Authoritarianism, psychopathy, and resistance to wearing masks during the COVID-19 pandemic: A partial replication and extension of key findings

**DOI:** 10.3389/fpsyg.2022.1049660

**Published:** 2023-01-18

**Authors:** Eric Charles Prichard, K. Amber Turner

**Affiliations:** School of Social and Behavioral Sciences, University of Arkansas at Monticello, Monticello, AR, United States

**Keywords:** COVID-19, authoritarianism, Machiavellianism, psychopathy, narcissism, personality, dark triad

## Abstract

Controversial recent research suggests Americans with more authoritarian tendencies showed less concern about COVID-19 and self-report less mask wearing. The present study set out to replicate this result with a larger sample. The study also sought to extend the original research by investigating how the Dark Triad traits of narcissism, psychopathy, and Machiavellianism are related 1 COVID-19 attitudes and behaviors. Nine-hundred ninety-six United States high school graduates were asked 8 questions about their level of concern about the effects of the virus on health and finances, how frequently they wore masks, which authority figures they trusted, and whether China was responsible for the virus. Correlational and regression analyzes showed that authoritarianism, Machiavellianism, narcissism, and psychopathy were all negatively related to self-reported mask wearing. An explanation is offered for an apparent contradiction between the presented results and studies that showed authoritarian support for lockdowns early during the outbreak.

## Introduction

An important activity in the scientific enterprise is the replication of noteworthy results. A largescale attempt to replicate psychological studies from top cited psychological journals found that results were replicated just under 50% of the time (Open Science Collaboration, 2015). This is concerning as, despite evidence that psychology as a discipline is starting to appreciate replication more than it has in the past, there is still a major bias in favor of publishing novel results (Nosek et al., 2022). The main purpose of this paper is to replicate a novel psychological result that received attention from some media sources during the height of the COVID-19 pandemic, to extend that result by considering other personality variables, and to replicate several other related findings.

Prichard and Christman (2020a) conducted a relatively small-scale study early in the pandemic asking participants how they rated various aspects of their level of concern about the virus, the extent to which participants trusted political figures and scientists, and the extent to which participants blamed China for the virus. They found that participants who scored higher on measures of authoritarian dominance, which is a measure of one’s willingness to actively impose a right-wing authoritarian worldview on others, were less concerned about the virus and reported less frequent mask wearing. They also blamed China more for the virus than people who score lower on the measure of authoritarian tendencies.

There have been several findings that suggest the results of Prichard and Christman’s (2020a) study may be more complicated than they first appear. Bochicchio et al. (2021) surveyed 757 Italian participants at the beginning of the pandemic and found that right-wing authoritarianism was associated with more maladaptive behavior. In the survey, maladaptive behaviors included xenophobic behavior toward Chinese immigrants in Italy and panic buying, both of which are consistent with Prichard and Christman’s results. However, several other maladaptive COVID behaviors were associated with excessive fear of the virus before there was an actual pandemic. This seems to contradict the lack of concern observed among Prichard and Christman’s American participants. Further complicating the picture, Manson (2020) found that measures of right-wing authoritarianism and left-wing authoritarianism both independently predicted endorsement of severe COVID-19 mitigation policies. Did something change in American’s perceptions and behaviors between the times Manson collected his data and Prichard and Christman collected theirs? Or are there additional variables which might be playing a key role?

One set of variables that prior research has connected to maladaptive COVID-related behaviors and attitudes is the Dark Triad consisting of psychopathy, Machiavellianism, and narcissism. For example, Triberti et al. (2021) found a negative association between COVID-19 mitigating behaviors and the traits of Machiavellianism and psychopathy in Italian participants. In a Polish sample, Nowak et al. (2020) found that those with higher levels of Dark Triad traits were likely to engage in more supply hoarding behaviors and less preventative behaviors. This finding was replicated recently in Brazil (Porfírio et al., 2023), while another Polish study found the Dark Triad was associated with less compliance with government mandates intended to curb the pandemic’s spread (Zajenkowski et al., 2020).

Interestingly, evidence from a study with a different Polish sample by Gogola et al. (2021) found differing results among the Dark Triad traits, with higher levels of psychopathy associated with disobeying pandemic recommendations while higher levels of narcissism predicted more compliance with these rules. A study with Spanish and Portuguese participants (Brito-Costa et al., 2022) similarly found that believing that people should listen to government recommendations was associated with less psychopathy and Machiavellianism, while narcissism was positively related to thinking people should listen to scientists. Yousaf et al. (2022) also found less maladaptive behavior associated with narcissism compared to Machiavellianism and psychopathy for panic buying in the face of scarcity. These findings point to the importance of considering each part of the Triad independently rather than as one combined factor.

Additionally, Ahadzadeh et al. (2021) found that the Dark Triad traits of Machiavellianism and narcissism weakened the association between healthy skepticism and resistance to online COVID-19 conspiracy theories. People high in psychopathy have been found to believe in COVID-19 conspiracy theories (March and Springer, 2019; Hughes and Machan, 2021). A number of studies have highlighted psychopathy’s link to risky behaviors related to the pandemic (e.g., Blagov, 2021; Doerfler et al., 2021; Konc et al., 2022).

Notably, while these findings from around the world indicate that the Dark Triad traits tend to be associated with less adaptive behaviors and attitudes related to COVID-19, results are not consistent for all three traits, highlighting the importance of analyzing each of the Dark Triad traits independently. The inconsistency among results may also point to a more complex relationship among variables, including the potential role that authoritarianism may play. As pointed out by Hardin et al. (2021), those high on Machiavellianism and narcissism may rely more on a stable social system to exploit and seek affirmation from others, respectively, which might draw them to more authoritarian beliefs.

Based on these findings, we might expect negative associations between reported concerns about the virus and mask wearing among people with high levels of psychopathy and Machiavellianism. Further, we might see associations between mistrust for scientific authorities and blaming China for the virus and the Dark Triad traits Machiavellianism and narcissism.

If the results of Prichard and Christman’s (2020a) study are replicated using a larger sample, then it is reasonable to be more confident that they accurately represent American attitudes. In addition to simple replication, we hope to make a novel theoretical and empirical contribution by including our measure of authoritarianism in regression models with a measure of the Dark Triad traits. Combining these traits with authoritarianism in our models may offer some hints that could solve the paradox: What made American authoritarians anti-authority when it came to wearing masks?

## Materials and methods

### Participants

Participants were 996 United States high school graduates who were recruited *via* Amazon’s Mechanical Turk (MTurk) data collection service. Research has shown that samples taken from MTurk provide a reasonable demographic match to the overall United States population in terms of gender and race, although MTurk samples do differ somewhat from the United States population in terms of religion (Burnham et al., 2018). As spirituality was not a primary topic of interest for the present study, we believe that the sample obtained will provided a reasonable approximation of the United States population for our purposes. Participants had a mean age of 43.9 years. Of the sample, 511 participants reported being female, 8 reported being non-binary, and 477 reported being male. Participants were also asked for their state of residence in order to make sure the sample represented a wide geographic range within the United States. While one cannot argue that an MTurk sample is perfectly representative of the United States population due to the self-selection involved in MTurk respondents choosing which surveys to participate in, our sample has an age range and geographic distribution which are wider than would be expected in a sample consisting solely of college students.

### Procedure and measures

Participants were recruited *via* a survey link in MTurk titled “COVID-19 and Personality.” If interested, they could read a description of the survey saying that they would be asked a series of questions about their personality and their experiences with COVID-19. The description also stated that the survey had been approved by the University of Arkansas at Monticello Institutional Review Board and that by clicking on the link and beginning the survey, participants were consenting to be part of the study. MTurk’s screening function was used to ensure that participants were United St high school graduates. Participants were asked to provide their age and state of residence and then completed the questionnaires. All items were shown in the same order for all participants. Upon completion of the study, participants were given a completion code. If they presented the appropriate completion code, participants were paid $1 within 24 h.

The participants completed the dependent measures first, which were taken directly from Prichard and Christman (2020a). These questions were used to gage attitudes about the impact of COVID-19 and virus-related health measures. Before the questions, participants received the instructions “On a scale of 1 to 7, with 1 being not at all concerned and 7 being very concerned, how concerned are you about the effects of COVID on yourself and others?” The questions were as follows:

1. “How worried and concerned are you about your personal health?”
2. “How worried and concerned are you about your financial situation?”
3. “How worried and concerned are you about the personal health of your fellow citizens?”
4. “How worried and concerned are you about the financial situation of your fellow citizens?”
5. “How often do you wear a mask when going out in public?”
6. “It is important to listen to and heed the advice given by experts and scientists.”
7. “It is important to listen to and heed the advice given by politicians and public figures.”
8. “China is directly responsible for the infection rates and death toll in the United States.”

All items were scored on seven-point scales. The first four were scored using the anchors “Not at all Concerned” and “Very Concerned” for extreme values. Item five used the anchors “Never” and “Always.” Items six through nine used the anchors “Strongly Disagree” and “Strongly Agree.”

Because a primary aim of this study was replication of Prichard and Christman’s (2020a) finding regarding authoritarianism and attitudes related to COVID-19, we measured authoritarianism using the same measure as that study, a modification of Rattazzi et al.’s (2007) short version of the Right-Wing Authoritarianism subscale. In other words, we used the seven-item “Authoritarianism Subscale,” which measures approval for using authoritarian tactics to impose a right-wing authoritarian value system (e.g., “Our country desperately needs a mighty leader who will do what has to be done to destroy the radical new ways and sinfulness that are ruining us.”). We also used the three optional “Authoritarian Submission” items that Rattazzi et al. (2007) list in their appendix. These items are: “The only way our country can get through the crisis ahead is to get back to our traditional values, put some tough leader in power, and silence the troublemakers spreading bad ideas,” “Once our government leaders give us the ‘go ahead’, it will be the duty of every patriotic citizen to help stomp out the rot that is poisoning our country from within,” and “What our country really needs is a strong, determined leader who will crush evil, and take us back to our true path.” Maintaining the scoring method used by Prichard and Christman (2020a), the Authoritarianism items were presented as seven-point scales anchored at 1 (Strongly Agree) and 7 (Strongly Disagree). For ease of interpretation, we reverse scored the entire scale prior to analysis so that higher scores would indicate more authoritarian attitudes.

Dark Triad traits were measured using a brief measure called the Short Dark Triad (SD3; Jones and Paulhus, 2014). It consists of 27 items measuring the traits of Machiavellianism (e.g., “It is not wise to tell your secrets”), Narcissism (e.g., “People see me as a natural leader”), and Psychopathy (e.g., “Payback needs to be quick and nasty”). All items were presented with five response options: Strongly Disagree, Disagree, Neither Agree or Disagree, Agree, and Strongly Agree. For the purpose of analysis, each response was recoded into a numerical score ranging from one to five, with a score of one indicating strong disagreement and a score of five indicating strong agreement. Each of the Dark Triad traits was scored as a separate scale and treated as a distinct predictor variable.

### Analysis and results

The analysis was conducted in four steps. In the first step, we calculated the means and standard deviations of all relevant variables. In the second step, we created a correlation matrix in which we looked at intercorrelations between the 4 predictor variables: authoritarianism, Machiavellianism, narcissism, and psychopathy. In the third step, we calculated correlation coefficients between the 4 predictor variables and the 8 COVID-19 attitude items. Because this step entails 32 comparisons, we used a Bonferroni correction to reduce the likelihood of making a Type I error. To do so, when investigating these raw correlations, we divided 0.05 (two-tailed) by 32 to calculate a significant *p*-value of 0.001. In the fourth step, we combined the 4 predictor variables and ran 9 regression models with each COVID-19 attitude item as a dependent variable. Because we ran 9 regressions, we divided.05 by 9, and only considered models with an overall model *p*-value of less than 0.0055 significant. However, when interpreting the independent contribution of the predictors within significant models, we considered *p*-value of less than.05. All *p*-values reported are based on 2-tailed significance. Based on the replicated study by Prichard and Christman (2020a), we made one *a priori* directional hypothesis: we expected authoritarianism to be correlated with less reported mask wearing and more blame for China. Other correlations and models were considered descriptive and exploratory investigations of the relationships between dark personality traits and COVID-19-related behaviors and attitudes.

Table 1 displays the relevant descriptive statistics. Table 2 displays the intercorrelations between the 4 predictor variables. Table 3 displays the correlations between the dark personality traits as well as authoritarianism and the 9 outcome variables.

**Table 1:** Descriptive statistics.

	*N*	Mean	Standard Deviation
How worried and concerned are you about your personal health?	996	4.69	1.847
How worried and concerned are you about your financial situation?	996	4.60	1.931
How worried and concerned are you about the personal health of your fellow citizens?	996	5.06	1.653
How worried and concerned are you about the financial situation of your fellow citizens?	996	4.95	1.598
How often do you wear a mask when going out in public?	996	5.94	1.866
It is important to listen to and heed the advice given by experts and scientists.	996	6.10	1.297
It is important to listen to and heed the advice given by politicians and public figures.	996	4.08	1.695
China is directly responsible for the infection rates and death toll in the United States.	996	3.47	2.151
Authoritarianism	996	32.82	17.639
Machiavellianism	996	23.72	5.743
Narcissism	996	22.84	6.407
Psychopathy	996	18.32	6.215

**Table 2:** Intercorrelation between predictor variables.

	Authoritarianism	Machiavellianism	Narcissism	Psychopathy
Authoritarianism	1.00	0.112**	0.195**	0.016
Machiavellianism	0.112**	1.00	0.387**	0.587**
Narcissism	0.195**	0.387**	1.00	0.424**
Psychopathy	0.016	0.587	0.424	1.00

**Significant at *p* < 0.001, our Bonferroni corrected value of *p*.

**Table 3:** Correlations between predictor and outcome variables.

	Authoritarianism	Machiavellianism	Narcissism	Psychopathy
How worried and concerned are you about your personal health?	0.036	0.023	0.008	−0.013
How worried and concerned are you about your financial situation?	0.075	0.132*	0.007	0.126*
How worried and concerned are you about the personal health of your fellow citizens?	−0.035	−0.106	0.024	−0.139*
How worried and concerned are you about the financial situation of your fellow citizens?	−0.069	−0.089	0.003	−0.068
How often do you wear a mask when going out in public?	−0.155*	−0.170*	−0.183*	−0.244*
It is important to listen to and heed the advice given by experts and scientists.	−0.197*	−0.088	−0.066	−0.165*
It is important to listen to and heed the advice given by politicians and public figures.	0.012	0.096	0.133*	0.046
China is directly responsible for the infection rates and death toll in the United States.	0.354*	0.172*	0.189*	0.100

*Significant at *p* < 0.001, our Bonferroni corrected *p*-value.

Authoritarianism was negatively associated with wearing masks in public and trusting scientific figures. It was positively associated with blaming China for the virus. This represents a replication of Prichard and Christman (2020a), but in a larger sample. As for the Dark Triad traits, Machiavellianism was positively associated with concern about one’s own finances and blaming China for the virus. It was negatively associated with self-reported frequency of mask wearing. This replicates a finding of Triberti et al. (2021) that Machiavellianism was associated with poorer COVID-preventative health behavior. Also consistent with Triberti, Durosini, and Pravettoni was the significant negative correlation between narcissism and frequency of masking. Narcissism was positively associated with trust for political figures and with blaming China for the virus. Psychopathy was negatively associated with concern about the financial situation of others, concern about the health of others, masking, and trust for scientists. It was positively associated with concern about one’s own financial wellbeing. All of the traits measured were negatively associated with wearing a mask. Each trait was significantly correlated with at least two other items. However, there are also modest correlations between all four of these traits.

The next question was whether each of these traits would independently account for variance in the outcome variables or whether one trait would emerge as a stronger correlate than the others. To assess this, for the next step we ran regression models using the four personality variables as predictor variables and the eight COVID-19 attitude response items as dependent variables. Tables 4–10 display our regression models. Only overall models with Bonferroni corrected *p*-values of less than.0055 are displayed.

**Table 4:** “How worried and concerned are you about your financial situation?” as outcome variable.

	*R*	*R* ^2^	*b*	SE	*β*	*t*	*p*
Overall model	0.178	0.032					<0.001
Authoritarianism			0.009	0.004	0.081	2.515	0.012
Machiavellianism			0.032	0.013	0.096	2.434	0.015
Narcissism			−0.027	0.011	−0.091	−2.547	0.011
Psychopathy			0.003	0.013	0.107	6.647	0.008

**Table 5:** “How worried and concerned are you about the personal health of your fellow citizens?” as outcome variable.

	*R*	*R* ^2^	*b*	SE	*β*	*t*	*p*
Overall model	0.154	0.024					<0.001
Authoritarianism			−0.004	0.003	−0.039	−1.218	0.224
Machiavellianism			−0.012	0.011	−0.043	−1.090	0.276
Narcissism			0.015	0.009	0.059	1.655	0.098
Psychopathy			−0.037	0.011	−0.138	−3.422	0.001

**Table 6:** “How worried and concerned are you about the financial situation of your fellow citizens?” as outcome variable.

	*R*	*R* ^2^	*b*	SE	*β*	*t*	*p*
Overall model	0.122	0.015					0.005
Authoritarianism			−0.006	0.003	−0.072	−2.211	0.027
Machiavellianism			−0.021	0.011	−0.077	−1.924	0.055
Narcissism			0.015	0.009	0.061	1.696	0.090
Psychopathy			−0.012	0.010	−0.048	−1.186	0.236

**Table 7:** “How often do you wear a mask when going out in public?” as outcome variable.

	*R*	*R* ^2^	*b*	SE	*β*	*t*	*p*
Overall model	0.293	0.086					<0.001
Authoritarianism			−0.015	0.003	−0.138	−4.440	<0.001
Machiavellianism			−0.002	0.012	−0.005	−0.134	0.893
Narcissism			−0.019	0.010	−0.064	−1.849	0.065
Psychopathy			−0.064	0.012	−0.212	−5.413	<0.001

**Table 8:** “It is important to listen to and heed the advice given by experts and scientists.” As outcome variable.

	*R*	*R* ^2^	*b*	SE	*β*	*t*	*p*
Overall model	0.261	0.068					<0.001
Authoritarianism			−0.015	0.002	−0.207	−6.572	<0.001
Machiavellianism			0.008	0.009	0.036	0.941	0.347
Narcissism			0.009	0.007	0.047	1.332	0.183
Psychopathy			−0.042	0.008	−0.203	−5.132	<0.001

**Table 9:** “It is important to listen to and heed the advice given by politicians and public figures.” As outcome variable.

	*R*	*R* ^2^	*b*	SE	*β*	*t*	*p*
Overall model	0.149	0.022					<0.001
Authoritarianism			−0.002	0.003	−0.022	−0.678	0.498
Machiavellianism			0.024	0.012	0.081	2.044	0.041
Narcissism			0.034	0.010	0.129	3.594	<0.001
Psychopathy			−0.015	0.011	−0.056	−1.381	0.168

**Table 10:** “China is directly responsible for the infection rates and death toll in the United States.” As outcome variable.

	*R*	*R* ^2^	*b*	SE	*β*	*t*	*p*
Overall model	0.386	0.149					
Authoritarianism			0.040	0.004	0.325	10.812	<0.001
Machiavellianism			0.039	0.014	0.104	2.810	0.005
Narcissism			0.029	0.011	0.087	2.591	0.010
Psychopathy			−0.001	0.013	−0.003	−0.092	0.926

Some interesting patterns emerge when the variables are combined. When the outcome variable was concern about finances, the traits of authoritarianism, Machiavellianism, and psychopathy were all independently positively correlated. Narcissism, which showed no relationship with this variable in the raw correlations, demonstrated a slight negative association with concern about one’s personal finances in the regression analysis. It is not immediately evident why this should be the case. One possibility is that people with more money, and thus more financial security, score higher on measures of narcissism. Another possibility is that the correlation could be spurious. As many of the other findings replicated previous research, we are fairly confident in them. This one was novel and somewhat unexpected, however. A line of future research may want to investigate the relationship between narcissism and peoples’ perception of their financial well-being during periods of economic distress.

Psychopathy stands out as the only variable with a significant negative association with concern about the personal health of other citizens; after psychopathy is entered in the model, the other variables fail to account for a significant amount of variance. Interestingly, it is only authoritarianism that has a significant negative association with concern about the financial wellbeing of others, while the other variables fail to account for significant variance in this outcome variable.

The masking item is somewhat intriguing. In the raw correlations, all four trait variables were negatively associated with frequency of wearing a mask. In the stepwise regression model, however, only authoritarianism and psychopathy show that association. Among the four personality variables examined in this study, they are the only two which are not correlated with each other. This suggests independent contributions of authoritarian personality traits and psychopathic personality traits in the resistance to wearing a mask.

Consistent with the above finding, authoritarianism and psychopathy are also the only variables which are negatively related to trusting the advice of scientists. Once again, each of these traits independently accounts for variance in the outcome variable. Only narcissism accounts for significant variance in the response to “It is important to listen to and heed the advice given by politicians and public figures.” Scores on the narcissism measure were positively associated with endorsement of this item. Finally, consistent with the raw correlations, authoritarianism, Machiavellianism, and narcissism were all positively associated with blaming China for the virus.

We will discuss limitations of the study in a general way at the end of the paper, but it is worth discussing a potential limitation of the analysis in the present section. In this paper, we treat the three Dark Triad traits and Authoritarianism as univariate independent predictors of the outcome variables. We also do not report any models including potential interaction effects, although we have uploaded our dataset to a publicly available open data platform if any readers are interested in exploring potential interactions. Our data can be found at: https://osf.io/zrjy4/.

## Discussion

One of the main goals of this study was to replicate Prichard and Christman’s (2020a) results showing that Americans who scored higher on a measure of authoritarian dominance tend to report masking with less frequency. We also found results consistent with previous studies showing that people who score higher on measures of Machiavellianism and psychopathy also performed less COVID-mitigating behaviors (Triberti et al., 2021). Our results associating narcissism and Machiavellianism with blaming China for the virus also corroborate prior findings that people who scored higher on these traits were more susceptible to COVID-related conspiracies (Ahadzadeh et al., 2021).

Another goal of this study was to address an apparent inconsistency in the literature. Earlier in the pandemic, Manson (2020) found that both individuals in a United States sample who scored high on measures of left-wing authoritarianism and those who scored high on right-wing authoritarianism were in favor of more stringent COVID-19 related measures. Bochicchio et al. (2021) found that people in an Italian sample who scored high on right-wing authoritarianism were more likely to take immediate and sometimes drastic protective measures, although they also showed more anti-Chinese xenophobia. By the time of Prichard and Christman’s (2020a) study, the United States sample they surveyed showed that people who scored high on a measure of authoritarian dominance reported being less apt to wear protective masks. The present study replicates that finding with a larger sample.

Why the apparent inconsistency? We propose that the inconsistency is just that: apparent. Gergen (1973) made the case that social psychology is a historical science because it measures people’s behavioral responses within a context. However, contexts like economic situations, political situations, cultural beliefs, and shared knowledge change. As such, behavioral responses might change, even within a relatively brief period of time. This can be frustrating for those of us who want psychology to find immutable laws and relationships in human behavior, but it could explain some of the difficulties with replicability in psychology as well as the paradox currently in question.

In the present study, our findings regarding the Dark Triad are consistent with findings from Italian (Triberti et al., 2021) and Malaysian (Ahadzadeh et al., 2021) samples. This consistency in results from different countries suggests that the Dark Triad traits are the kind of stable personality traits that may allow researchers to generalize across cultural or political contexts. Psychopathy and Narcissism are related to general self-absorption and lack of concern about others. For example, one of the items in the SD3’s Psychopathy subscale asks about enjoyment of sex with strangers. This is an item that measures sensation seeking, risk taking, and potentially putting others at risk in order to engage in a sensation seeking activity. It is not hard to imagine someone who scores high on this measure deciding to forego a mask that might be mildly uncomfortable because that immediate comfort and sensation will outweigh concern for others.

Authoritarian dominance, however, is a slightly more complicated to interpret. The measure used in this study and in Prichard and Christman (2020a) was also used by Prichard and Christman (2020b) in a completely unrelated study surveying voters from the 2016 United States primaries. In that study, the same authoritarian dominance measure was related to support for Donald Trump among Republican primary voters. Béland et al. (2021) conducted a case study of the presidential responses of Donald Trump and Jair Bolsonaro, the right-leaning populist president of Brazil, to COVID-19. The authors point out that both presidents have authoritarian and anti-democratic tendencies and both publicly downplayed the significance of the viral threat to their respective countries’ detriments.

Although the study is correlational, we still want to offer some potential explanations for why the followers of authoritarian leaders might have been more apt to oppose COVID measures. One possibility to consider is that Dark Triad traits may be best conceptualized as part of a single latent factor when correlated with high-level trait variables such as a sociocultural variable. The second table shows that there is a large degree of intercorrelation among the predictor variables. Jonason et al. (2011) did a series of studies in which they found that the three Dark Triad traits were more useful as independent correlates when the outcome variables were mid-level traits such as traits related to interpersonal manipulation of romantic partners, but the Dark Triad traits were more useful as components of a single latent factor when the outcome variable was a high-level trait like sociocultural attitudes. We would argue that the outcome variables in the present study are closer to the interpersonal mid-level variables that are better predicted by a three-trait model. However, Authoritarianism is a higher-level sociocultural variable that might be better predicted by a latent Dark Triad factor. If this is the case, it is worth considering whether the Dark Triad traits are part of a latent emotional instability factor that leads people to find authoritarian leadership styles more appealing. It is possible that the latent variable is emotional dysregulation associated with self-serving, aggressive, and interpersonally destructive behaviors. Authoritarian leadership rationalizes such behavioral patterns as long as they can be construed as being beneficial for the “nation” or the “people,” however such an amorphous group is defined. Whether Authoritarians are in favor of imposing strict lockdowns on others or actively harassing public health officials who are in favor of virus mitigations measures will depend on what entity the perceived and trusted authority labels as the enemy. Authoritarianism justifies the dysregulated behaviors related to the Dark Triad latent factor, as long as those behaviors are directed toward the correct “bad guy.”

What we propose is that one cannot simply separate a personality variable such as authoritarianism from the present social context of the sample from which the measure was taken. We often think of disciplines such as psychology or political science as siloed and separate from each other. But what if the effect of a psychological variable is dependent on the political context? In an American context, people with tendencies toward authoritarian dominance were attracted to a candidate with similar traits. When that candidate, as president, downplayed the virus, this likely led to his supporters downplaying the virus and following his lead in mistrusting scientists. However, earlier in the pandemic, people with both left- and right-wing authoritarian tendencies were apt to support restrictive COVID measures (Manson, 2020). Had leaders such as Trump in the United States or Bolsonaro in Brazil advocated for strong measures and endorsed the authority of scientists, this might well have continued to be the case.

The point is not that it would have been good if authoritarians had been in favor of strong COVID-19 measures. Instead, our point is that authoritarianism as variable was related to attitudes and behaviors in response to COVID-19, but those attitudes and behaviors differed from place to place and from early in the pandemic to later in the pandemic. Gergen (1973) may have been at least partially correct when he argued that psychology is a historical science. If this is the case, then psychologists studying personality should consider more interdisciplinary research with social scientists who are expert in a particular social or political context when using personality-based research to make recommendations about topics of broad public interest such as public health.

Finally, we want to address some limitations of the present study. Participants were recruited using MTurk, which by its nature is not a truly representative sample. Furthermore, we restricted our sample to people who reported being graduates of United States high schools. Results may have varied if we had chosen a more restrictive sample, such as only college graduates, or alternately a more diverse sample that included people who did not finish high school. As most studies, ours included, have assessed attitudes and behaviors within one country, multinational research on this topic could be especially beneficial (e.g., Brito-Costa et al., 2022). Our variables were cross-sectional and measured *via* self-report. In future research on how people respond to public health measures, a more ecologically valid measure of behavior might be warranted. This could include using phone surveys to ask people in high outbreak areas whether they are currently taking preventative measures. A last limitation is the relative simplicity of the analysis. We use linear regression and treat the three Dark Triad traits as separate traits. However, we recommend that future research consider using latent variable modeling methods and compare a three trait Dark Triad model to a single latent factor Dark Triad model, especially regarding the relationship between the Dark Triad and Authoritarianism.

### Statement of significance

This paper is of broad interest to the reading public and psychological scientists because it provides evidence that common personality traits interact with social and political factors to affect health behaviors in a global pandemic setting. After more than two years of struggling with a global pandemic, it had become clear that individual differences predict who is more likely to take precautions. They also predict susceptibility to messages which may hinder public health efforts. By understanding how the psychology of personality affects behavior in pandemics, future public health officials may develop more effective and nuanced messaging strategies when the next pandemic hits.

## Data availability statement

The original contributions presented in the study are publicly available. This data can be found here: https://osf.io/zrjy4/.

## Ethics statement

The studies involving human participants were reviewed and approved by University of Arkansas at Monticello Human Subjects Institutional Review Board. Written informed consent for participation was not required for this study in accordance with the national legislation and the institutional requirements.

## Author contributions

EP and KT both conceived of the study and pick the instruments. EP collected the data and wrote the first draft. KT revised the draft and produced the second draft. All authors contributed to the article and approved the submitted version.

## Funding

This research was partially funded by an institutional research grant from University of Arkansas at Monticello.

## Conflict of interest

The authors declare that the research was conducted in the absence of any commercial or financial relationships that could be construed as a potential conflict of interest.

## Publisher’s note

All claims expressed in this article are solely those of the authors and do not necessarily represent those of their affiliated organizations, or those of the publisher, the editors and the reviewers. Any product that may be evaluated in this article, or claim that may be made by its manufacturer, is not guaranteed or endorsed by the publisher.
